# Immediate Effect of Anterior Cruciate Ligament Protective Knee Taping on Knee Landing Mechanics and Muscle Activations during Side Hops

**DOI:** 10.3390/ijerph181910110

**Published:** 2021-09-26

**Authors:** Yu-Lun Huang, Kuang-Wei Lin, Li-Wei Chou, Eunwook Chang

**Affiliations:** 1 Department of Physical Education and Sport Sciences, College of Sports and Recreation, National Taiwan Normal University, Taipei 11677, Taiwan; huanyulu@ntnu.edu.tw; 2 Department of Physical Therapy and Assistive Technology, National Yang Ming Chiao Tung University, Taipei 11221, Taiwan; wade1112@gm.ym.edu.tw (K.-W.L.); lwchou@nycu.edu.tw (L.-W.C.); 3 Department of Kinesiology, Inha University, Incheon 22212, Korea

**Keywords:** injury prevention, external knee support, neuromuscular control

## Abstract

Athletic taping is widely used in sports to prevent injury. However, the effect of anterior cruciate ligament (ACL) protective taping on neuromuscular control during dynamic tasks remains unclear. Therefore, this study aimed to investigate the immediate effect of ACL protective taping on landing mechanics and muscle activations during side hops in healthy individuals. Fifteen healthy individuals (11 males and 4 females; age, 23.1 ± 1.4 years; height, 175.1 ± 10.4 cm; weight, 66.3 ± 11.2 kg) volunteered to participate in this study. Landing mechanics and muscle activations were measured while each participant performed single-leg hops side-to-side for ten repetitions with and without taping. An optical motion capture system and two force plates were used to collect the kinematic and kinetic data during the side hops. Surface electromyogram recordings were performed using a wireless electromyography system. Paired t-tests were performed to determine the differences in landing mechanics and muscle activations between the two conditions (taping and non-taping). The level of significance was set at *p* < 0.05. Compared with the non-taping condition, participants landed with a smaller knee abduction angle, greater knee external rotation angle, and smaller knee extensor moment in the taping condition. Given that greater knee abduction, internal rotation, and knee extension moment are associated with a greater risk of ACL injury, our findings suggest that ACL protective taping can have an immediate effect on dynamic knee stability. Clinicians should consider using ACL protective taping to facilitate the use of favorable landing mechanics for ACL injuries.

## 1. Introduction

Anterior cruciate ligament (ACL) injuries are common in sports, as approximately 91% of ACL injuries occur during sporting activities [1]. At least two-thirds of ACL tears are non-contact injuries while athletes perform jumping, cutting, or pivoting with unfavorable landing mechanics, including greater knee abduction coupled with increased rotation of the tibia and increased quadriceps muscle activation [2,3]. Hewett et al. [4], in a prospective study, observed that female athletes who went on to experience a non-contact ACL injury exhibited an 8° greater knee abduction, 2.5% greater internal knee adduction moment, 20% higher ground reaction force (GRF), and 16% shorter stance time during landing tasks, compared to athletes without subsequent ACL injury. Additionally, Hewett et al. [4] reported that excessive knee abduction movement and internal knee adduction moments during landing are predictors of ACL injury risk in female athletes.

In addition to the landing mechanics associated with ACL injury risk in the frontal and transverse planes, landing with greater knee extensor moment at a more extended knee joint angle has also been shown to be a risk factor associated with ACL injuries [5,6]. The quadriceps muscle is the primary active stabilizer of the knee joint during dynamic tasks. However, previous studies have suggested that an ACL strain is greatly contributed to by excessive quadriceps contraction, especially while landing with a more extended knee position [6,7]. Besides the quadriceps muscles, other surrounding muscular structures also influence the stress placed on the ACL. For instance, the gastrocnemius muscle acts as an ACL antagonist when the knee joint is near full extension [8]. In contrast, hamstring muscles provide a counterbalancing force to protect the ACL by minimizing anterior tibial translation and internal rotation induced by quadriceps contraction [9,10]. Hence, it is essential to identify interventions that can facilitate the use of safer landing mechanics and favorable muscle activations to minimize the risk of ACL injury.

Protective taping is widely used in sports to prevent injury [11]. Although the effects of taping on patellofemoral pain syndrome and ankle sprains have been investigated in previous studies [12,13,14,15], the effect of ACL protective taping and wrapping techniques, which have been used clinically to prevent ACL injuries by restricting excessive knee joint movements [16], have not been fully explored during functional movements. A study that used a cadaveric model to study the mechanical effect of external knee supports reported that ACL protective taping and functional knee braces provide a mechanical effect on reducing knee anterior-posterior translation and internal–external rotation [17]. However, this cadaveric model study did not account for the potential influence of muscle activation. Furthermore, the results of the cadaveric model study may not be generalized to dynamic activities because they do not directly reflect the risk of ACL injury biomechanically. The effects of ACL protective taping on landing mechanics and muscle activations during functional tasks need to be assessed. Therefore, this study aimed to investigate the immediate effects of ACL protective taping on landing mechanics and muscle activations during side hops in healthy individuals. We hypothesized that during the taping (TP) condition, individuals would demonstrate more favorable landing mechanics and muscle activations than the non-taping (NTP) condition, such as larger hamstring muscle activation, lesser GRFs, lesser knee abduction and internal rotation angles, and lesser knee extensor and abductor moments.

## 2. Materials and Methods

### 2.1. Participants

Sample size estimation was conducted based on the results reported by a previous study [18] with a power of 80% and a two-sided test with a significance level of 0.05. According to our a priori power analysis, 11 participants were needed to statistically detect the mean knee joint moment differences of individuals before (1.61 ± 0.19 Nm/kg) and after (1.43 ± 0.18 Nm/kg) patellar taping [18]. Accounting for potential dropouts, 15 healthy individuals (11 male and 4 female; age, 23.1 ± 1.4 years; height, 175.1 ± 10.4 cm; weight, 66.3 ± 11.2 kg) without lower extremity dysfunction, surgery, or joint injury in the past 12 months were recruited. All participants were active recreational individuals who participated in moderate physical activity for at least 150 min per week. All participants underwent an initial screening, and their dominant limbs were identified. The leg that would be used to kick a ball as far as possible was identified as the dominant lower limb [4]. All testing procedures were approved by the Institutional Review Board of National Yang-Ming University and written informed consent was obtained from the participants before the beginning of data collection.

### 2.2. Instrumentation

The Vicon T40 motion analysis system (Vicon Motion Systems Ltd., Oxford, UK) with 10 infrared cameras to capture the three-dimensional trajectories of the reflective markers with a sampling rate of 100 Hz was used. Two force platforms (Type 9287A and 9281B, Kistler Instrument Corp., Winterthur, Switzerland) were used for synchronically recording the GRFs during side hops. The analog force signal was sampled at 1000 Hz, analog-to-digital converted at 12-bit resolution, and stored on a computer. Vertical GRF above and below the threshold of 10 N was used to determine the initial contact and take-off, respectively. An eight-channel wireless surface electromyography (EMG) system (BTS Engineering, Bologna, Italy) with a sampling rate of 1000 Hz was used to record muscle activations synchronically with the Vicon motion capture system during side hops.

### 2.3. ACL Protective Taping Technique

**Figure 1:**
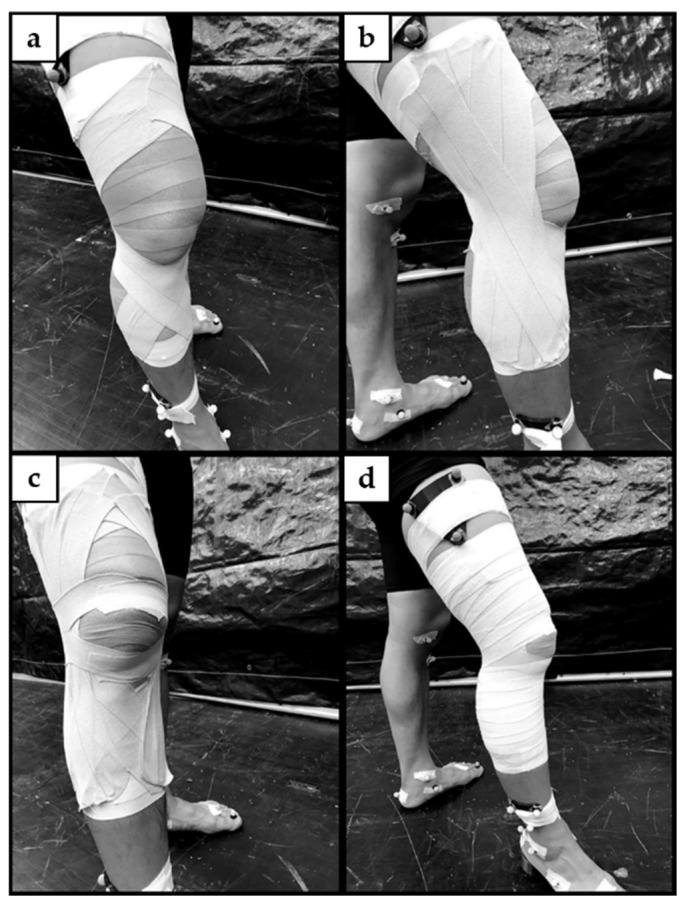
Anterior cruciate ligament protective taping techniques comprise three parts: (**a**) Rotatory taping strips. (**b**) Collateral ligament knee taping strips. (**c**) Patella taping strips. (**d**) Covered with 2-inch elastic adhesive light wrap tape from the bottom to the top.

A standard ACL protective knee taping technique described by Perrin [19] was applied by the same certified athletic trainer. The participant’s skin was cleaned and dried before taping. Open wounds were covered as required. Participants were instructed to position the dominant leg in front and step on a 1.5-inch heel lift at a slight knee flexion angle while shifting their body weight to the front leg (Figure 1). Two lace pads coated with a friction-reducing cream were placed over the tendons of the hamstring muscles. A single layer of pre-wrap was applied from the mid-tibia to the mid-thigh. Proximal and distal anchors were applied on the ends of the pre-wrap with a 1.5-inch nonelastic adhesive white tape (Johnson & Johnson, New Brunswick, NJ, USA), attached directly to the skin to prevent unwanted gliding during dynamic movements. ACL protective taping techniques comprise three parts. Rotatory strips (Figure 1a) were applied, followed by collateral ligament knee taping strips (Figure 1b) and patella taping strips (Figure 1c) with 3-inch elastic tape (Johnson & Johnson, New Brunswick, NJ, USA). Lastly, a 2-inch elastic adhesive light wrap tape was used for covering the distal to proximal part, completed with two locking strips with a 1.5-inch nonelastic adhesive white tape at both ends (Figure 1d).

### 2.4. Experimental Procedures

**Figure 2:**
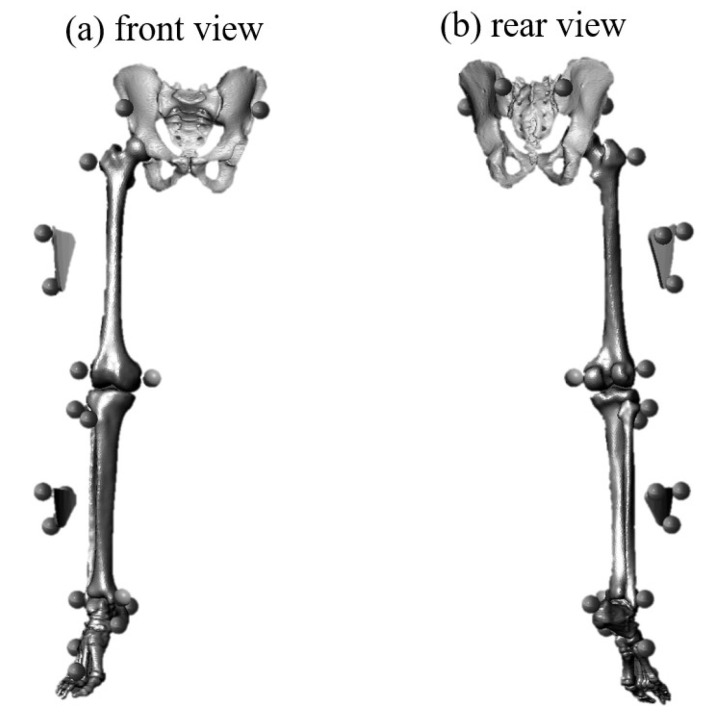
(**a**) Frontal view and (**b**) rear view of reflective marker placement.

Prior to data collection, participants completed a 20 min standard warm-up protocol, including 10 min of jogging or stationary cycling, followed by squats, toes raises, and light jumps (two sets of 10 repetitions). After the warm-up, 21 reflective markers were placed bilaterally on the anterior and posterior superior iliac spines, greater trochanter, lateral and medial femoral epicondyles, head of the fibula, tibial tuberosity, lateral and medial malleolus, heel, navicular tuberosity, and the tuberosity of the second and fifth metatarsals (Figure 2). Additionally, two cluster sets that were built with three non-collinear markers were affixed to the upper portion of the thigh and lower portion of the shank, which were not covered by the ACL protective taping. 

The EMG electrodes were attached bilaterally following the reflective marker placements. Before EMG electrode placements, the participant’s skin was shaved and cleaned with alcohol pads. Electrodes for each targeted muscle were attached to the muscle belly, longitudinally along the orientation of the muscle fibers with a standard distance (2 cm) between electrodes. The muscle activities of the rectus femoris, vastus lateralis, vastus medialis, semitendinosus, biceps femoris, lateral gastrocnemius, and medial gastrocnemius muscles were recorded during side hops.

**Figure 3:**
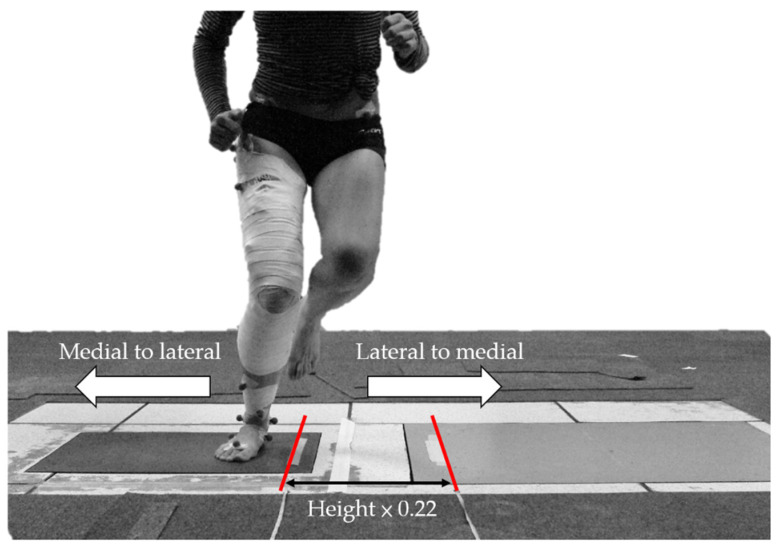
The participants were asked to preform single-leg hops side to side between two parallel lines. The distance between the two parallel lines was the product of participant’s height and 0.22.

The participants were given a 5-min practice trial to familiarize themselves with the testing movement. Participants were instructed to stand barefoot on the dominant leg and perform single-leg hops side-to-side as fast as possible between two parallel lines (10 repetitions). Successful trials were defined as participants hopping continuously and landing completely on a force plate without a pause or their foot slipping during landing. Three successful trials during each condition were collected with at least a 3-min break between trials to prevent fatigue. During testing, the TP condition was followed by the NTP condition. The distance between two parallel lines in the study conducted by Gustavsson et al. was 40 cm [20]. However, during our pilot study, we found that 40 cm was too wide for shorter participants. Thus, to account for the variability of height among participants, the distance between the two parallel lines in the current study was calculated based on the participant’s height. According to the participants’ demographic information in the study by Gustavsson et al. [20], the mean height of the male group was 181 ± 8 cm. We divided 40 cm by 181 cm (approximately 0.22) as a constant to calculate the distance between two parallel lines for each participant. The distance between the two parallel lines during the side hops was the product of the participant’s height and 0.22 (Figure 3).

### 2.5. Data Reduction

All data were processed using a custom-written software program (MATLAB 2020a; MathWorks Inc., Natick, MA, USA). Marker data were filtered using a fourth-order zero-lag Butterworth 10 Hz low-pass filter. A body-embedded orthogonal coordinate system was defined for each body segment using the markers, with the positive x-axis directed anteriorly, the positive y-axis superiorly, and the positive z-axis directed to the right. A static reference trial was recorded to identify the locations of the markers surrounding the knee joint, including the tibial tuberosity, fibular head, and both medial and lateral epicondyles, with respect to the cluster markers, and to define the neutral joint angles in the anatomical position. After the static reference trial, the markers on the tibial tuberosity, fibular head, and medial and lateral epicondyles were removed. Joint angles were calculated using the z-x-y Cardan rotation sequence. The knee joint center was determined by the midpoint between the medial and lateral epicondyles of the femur. The Newton–Euler equations of motion were used to calculate the joint moments. GRFs were normalized by body weight, and joint moments were normalized by body weight × leg length. Leg length was defined as the distance between the anterior superior iliac spine and the medial malleolus during the static trial.

The side hop motion comprises two parts: lateral-to-medial hop and medial-to-lateral hop. In the current study, the lateral touchdown phase was defined as hopping from the medial (close to the midline of the body) to the lateral. For example, when the testing leg is the right leg, the lateral touchdown phase comprises hopping from left to right. Since the knee adduction load applied to the knee during the lateral touchdown phase of the side hop was larger than the medial touchdown phase [21], only the lateral touchdown phase was analyzed in our study. The kinematic and kinetic data of each trial were interpolated to 101 points during the braking (from initial contact to maximum knee flexion) and propulsion (from maximum knee flexion to take-off) phases for time normalization via cubic spline interpolation. After interpolation and time normalization, to translate the movement cycle into percentage data, the kinematic and kinetic data were averaged at 10% intervals across three trials to construct a representative pattern for each condition. 

Raw EMG signals were filtered with a bandwidth filter between 10 Hz and 500 Hz using a custom-written software program (MATLAB 2020a; MathWorks Inc., Natick, MA, USA), and smoothed using a symmetrical moving root mean square (RMS) filter (20 ms time constant). The amplitude of the RMS EMG was calculated for the braking and propulsion phases. The peak amplitudes during the braking and propulsion phases represent 100% muscle activity, and the average RMS data during each phase are expressed as a percentage of the peak RMS [22].

### 2.6. Statistical Analysis

For statistical analysis, ground contact time (from initial contact to take off), peak vertical GRF, anterior and posterior GRFs, time to peak vertical GRFs, RMS muscle activation, knee joint range of motion (ROM), and internal joint moments were averaged across three trials. The knee joint angle and joint moment profiles were ensemble-averaged across all participants for the NTP and TP conditions. Means and standard deviations at 10% increments during each phase were obtained, and separate paired t-tests were conducted for comparisons between conditions with a significance level of 0.05. The MATLAB software was used for statistical analyses.

## 3. Results

### 3.1. GRFs

No significant difference was observed in the ground contact time, time to peak vertical GRF, and peak vertical, anterior, and posterior GRFs between the NTP and TP conditions (Table 1).

### 3.2. Muscle Activation

**Figure 4:**
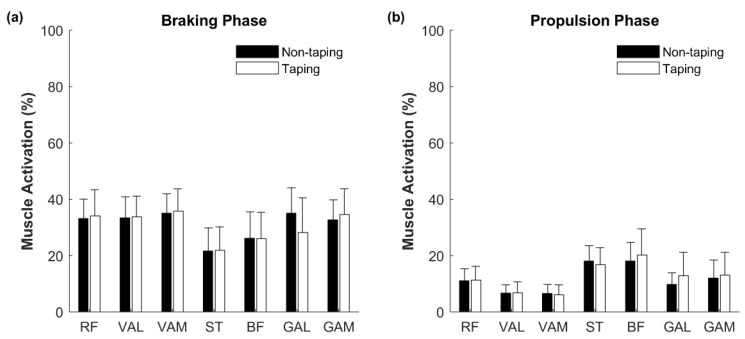
Muscle activation during side hops. (**a**) The electromyography (EMG) activity level of the examined muscles in the braking phase. (**b**) The EMG activity level of the examined muscles in propulsion phases. RF: rectus femoris; VAL: vastus lateralis; VAM: vastus medialis; ST: semitendinosus; BF: biceps femoris; GAL: lateral gastrocnemius; GAM: medial gastrocnemius.

No significant difference in muscle activation was observed for any muscle group during the braking and propulsion phases (Figure 4).

### 3.3. Knee Joint Angles

**Figure 5:**
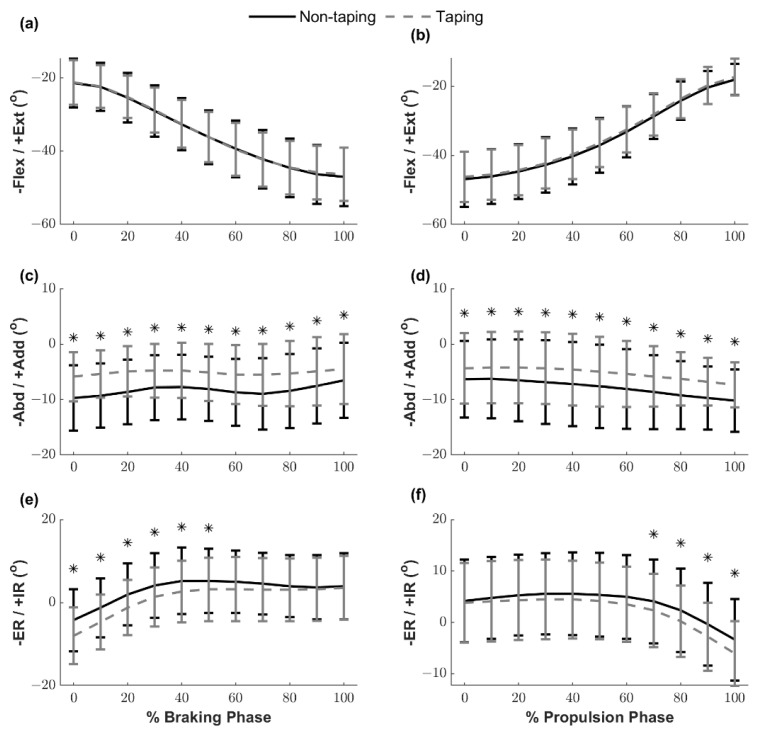
Knee joint angle ensemble curves of the braking and propulsion phases, shown for 10% increments. (**a**) Knee flexion/extension angle in the braking phase; (**b**) knee flexion/extension angle in the propulsion phase; (**c**) knee abduction/adduction angle in the braking phase; (**d**) knee abduction/adduction angle in the propulsion phase; (**e**) knee external rotation/internal rotation angle in the braking phase; (**f**) knee external rotation/internal rotation angle in the propulsion phase. * Significant differences between the conditions. Flex/Ext: flexion/extension; Abd/Add: abduction/adduction; ER/IR: external rotation/internal rotation.

The ensemble curves of the knee joint angle during the braking and propulsion phases are illustrated in Figure 5. The knee flexion–extension joint angles were not different between the NTP and TP conditions during the braking and propulsion phases. Knee abduction angles during the TP condition at each time point were significantly smaller than the NTP condition in both phases (*p* < 0.001–0.006). Furthermore, the knee external rotation angle during the TP condition was significantly greater than the NTP condition in the first half of the braking phase (from initial contact to 50%) (*p* = 0.002–0.023) and after 70% of the propulsion phase to take off (*p* = 0.005–0.038).

The comparisons of knee ROM of the braking and propulsion phases during side hops between the two conditions are presented in Table 2. Significant differences in the knee ROM between the two conditions were found in the frontal plane during the braking phase and in the transverse plane during the propulsion phase.

### 3.4. Knee Joint Moments

**Figure 6:**
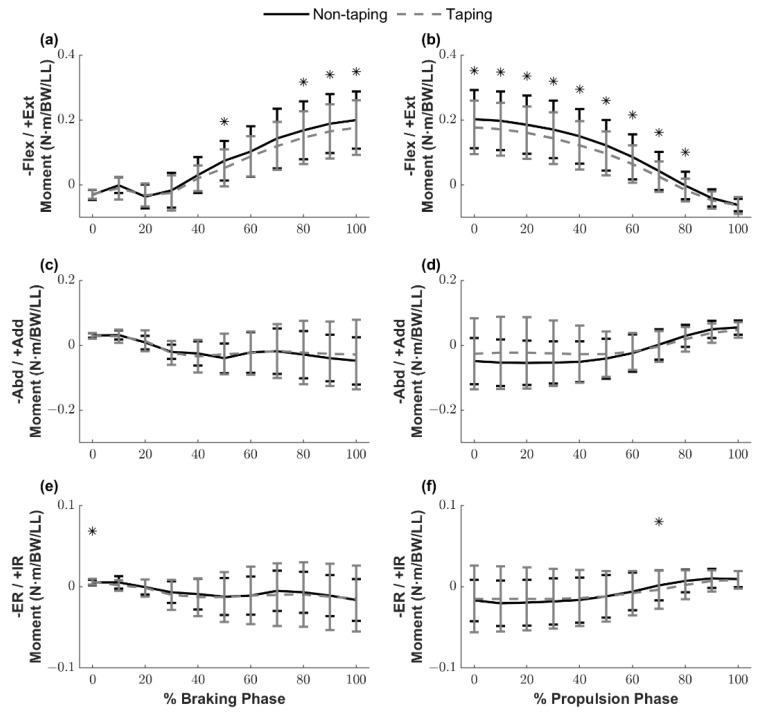
Knee joint moment ensemble curves during the braking and propulsion phases, shown for 10% increments. (**a**) Knee flexion/extension joint moment in the braking phase; (**b**) knee flexion/extension joint moment in the propulsion phase; (**c**) knee abduction/adduction joint moment in the braking phase; (**d**) knee abduction/adduction joint moment in the propulsion phase; (**e**) knee external rotation/internal rotation joint moment in the braking phase; (**f**) knee external rotation/internal rotation joint moment in the propulsion phase. * Significant differences between the conditions. Flex/Ext: flexor/extensor; Abd/Add: abductor/adductor; ER/IR: external rotator/internal rotator; BW: body weight; LL: leg length.

The ensemble curves of the knee joint moments are illustrated in Figure 6. Knee extensor moments in the TP condition were significantly smaller than those in the NTP condition at 50%, 80%, 90%, and 100% of the braking phase (*p* = 0.024–0.034), and from 0% to 80% of the propulsion phase (*p* = 0.010–0.043). Although no significant difference was found in knee adductor/abductor moments, there was a significantly greater internal rotator moment at initial contact (0%) of the braking phase (*p* = 0.036) and a smaller internal rotator moment at 70% of the propulsion phase in the TP condition (*p* = 0.042).

## 4. Discussion

This study aimed to investigate the effects of ACL protective taping on landing mechanics and muscle activations during side hops in healthy individuals. The primary finding of this study is that ACL protective taping successfully provided frontal plane stability by decreasing the knee abduction angle during side hops. We observed that ACL protective taping maintained at a reduced knee abduction angle (around 5°) throughout the braking and propulsion phases slightly reduced the knee frontal plane ROM during the braking phase by approximately 1.4°. During the initial impact phase, while the knee joint was in a more extended position (flexed < 35°), the knee internal/external rotation ensemble curves significantly shifted toward a more external rotation direction under the TP condition. Lastly, the knee transverse plane ROM increased slightly by approximately 1.2° during the propulsion phase.

A previous study suggested that ankle protective taping decreases ankle inversion, resulting in 37–61% of available ankle ROM [23]. In the current study, we observed that ACL protective taping facilitated a greater external rotated knee position while landing and maintained the knee joint at a reduced knee abduction position throughout (from initial contact to take off) and decreased the knee frontal plane ROM during the braking phase compared with the NTP condition. In contrast, ACL protective taping did not restrict knee joint movement in the sagittal plane. These findings indicate that ACL protective taping provides dynamic knee joint stability in the frontal and transverse planes, preventing ACL injury by facilitating the use of more favorable landing mechanics. More importantly, ACL protective taping did not restrict the shock absorption mechanism in the sagittal plane in healthy individuals. Kim et al. [24] investigated the effect of rigid and elastic taping on knee joint alignment during a golf swing. They applied rigid and elastic taping techniques to provide lateral stability to the lead leg and aimed to correct joint alignment by controlling tibial rotation against the femur and observed that rigid taping successfully reduced the knee varus angle of the lead leg. In the current study, the ACL protective taping technique comprised three parts, the collateral ligament knee taping strips aimed at providing support and stability in the frontal plane, which is similar to the rigid taping technique used in Kim’s study [24].

Knee abduction motion coupled with internal tibial rotation is associated with a higher ACL injury risk, given that a video analysis of ACL injuries that utilized model-based image-matching techniques showed that ACL injuries occurred shortly after initial contact, with an average 12° increase in the knee abduction angle and an 8° increase in the internal rotation angle [25]. Moreover, landing with a reduced knee flexion position accompanied by excessive knee internal rotation, abduction loading, and quadriceps muscle contraction during sudden decelerating tasks may induce ACL injuries by increasing ACL loading [26]. Consistent with our hypothesis, during the TP condition, our participants demonstrated smaller knee abduction motion (joint angle and total ROM) and reduced knee internal rotation with a similar knee flexion/extension pattern, compared with the NTP condition. These modifications of knee kinematics in the frontal and transverse planes suggest that the ACL protective taping technique facilitates the use of more favorable landing mechanics with regard to ACL injury risk, which potentially mitigates the risk of ACL injury.

Anterior tibial shear force contributed to by an aggressive quadriceps contraction near full knee extension, and a lack of hamstring muscle activation may collectively increase the risk of ACL injury [5,6,10]. Our study reported that, in the TP condition, knee extensor moments during the braking phase while the knee flexed greater than nearly 35° were significantly smaller than the NTP condition. ACL injuries have been reported to occur approximately 40 ms after initial contact [25]. In the current study, knee extensor moments were not influenced by ACL protective taping during this critical time window (within 40 ms after initial contact, approximately 0–20% of the braking phase). These findings suggest that ACL protective taping did not negatively affect knee moments in the sagittal plane when the knee was still at a reduced knee flexion position (<35°) while providing dynamic stability in the frontal and transverse planes.

Our original hypothesis was that ACL protective taping could decrease knee extensor moments due to an increase in hamstring muscle activation. The results of our study showed that ACL protective taping reduced peak knee extensor moments. However, no significant differences were identified in the quadriceps, hamstrings, and gastrocnemius muscle activation levels. These unexpected findings may indicate that there could be an additional knee flexor moment resulting in the tensile forces of the elastic tape while the knee flexed over 35° rather than a change in the hamstring muscle activation leading to a smaller net knee extensor moment. Future studies are required to examine this notion.

A simulation study reported that a combination of abduction and internal rotation moments applied on a knee joint during landing significantly increased the peak ACL strain [27]. In the current study, we observed that ACL protective taping increased the external rotation moment significantly at initial contact; however, the values were trivial and must be interpreted cautiously because the clinical significance is unclear. Lastly, although ACL protective taping failed to alter the abduction moment, it facilitated the use of safer frontal plane landing mechanics during side hops by maintaining reduced knee abduction angles throughout the braking and propulsion phases. Accordingly, our kinematics and kinetics findings indicate that ACL protective taping provides dynamic knee stability primarily by minimizing the frontal plane knee joint ROM and abduction angle without altering frontal plane knee joint moments.

Contrary to our hypothesis, ACL protective taping failed to lower the peak vertical, posterior, and anterior GRFs. A potential explanation for this finding is that as the sagittal plane knee joint angles and ROM were not different between the NTP and TP conditions, ACL protective taping did not compromise the shock absorption mechanism during landings [28]. This result is consistent with our previous work investigating the influence of protective taping in ACL-deficient individuals [29].

Our study has some limitations. First, we were unable to quantify the ACL protective taping strain during dynamic movements. However, we do not believe that the varied ACL protective taping strain affected our findings because the ACL protective taping was applied by the same certified athletic trainer for every participant to ensure that the ACL protective taping was standardized. Second, this study only investigated the immediate effects of ACL protective taping in individuals without ACL injuries. Future studies are needed to examine whether ACL protective taping can provide similar protective effects in individuals with ACL-reconstructed knees.

## 5. Conclusions

In conclusion, the results of the current study suggest that ACL protective taping techniques successfully provide knee joint dynamic stability in the frontal and transverse planes without compromising the sagittal plane shock absorption mechanism. ACL protective taping did not alter the magnitude of peak GRFs or muscle activations. Thus, ACL protective taping techniques facilitate the use of more favorable knee landing mechanics. Clinicians should consider using ACL protective taping techniques to facilitate the use of more favorable knee landing mechanics with respect to ACL injury risk.

## Figures and Tables

**Table 1:** Ground contact time, peak GRFs, and time to peak vertical GRF.

	Conditions (Mean ± SD)		
Variables	Non-Taping	Taping	*p*-Value
Ground contact time (s)	0.34 ± 0.14	0.35 ± 0.16	0.76
Time to peak vertical GRF (s)	0.16 ± 0.04	0.16 ± 0.05	0.69
Peak vertical GRF (BW)	2.47 ± 0.27	2.48 ± 0.37	0.94
Peak anterior GRF (BW)	0.27 ± 0.09	0.24 ± 0.08	0.20
Peak posterior GRF (BW)	0.18 ± 0.09	0.17 ± 0.08	0.81

SD: standard deviation; GRF: ground reaction force; s: second; BW: body weight.

**Table 2:** Knee ROM of Braking and Propulsion Phases in Three Different Planes.

	Condition (Mean ± SD)			
ROM (°)	Non-Taping	Taping	*p*-Value	Effect Size
Braking phase				
Sagittal plane	26.09 ± 6.12	25.40 ± 5.94	0.30	0.11
Frontal plane	6.54 ± 2.30	5.13 ± 2.16	0.01 *	0.63
Transverse plane	12.33 ± 4.93	13.43 ± 5.17	0.19	0.22
Propulsion phase				
Sagittal plane	30.31 ± 6.10	29.80 ± 5.73	0.65	0.09
Frontal plane	5.95 ± 2.44	5.38 ± 2.09	0.22	0.25
Transverse plane	10.52 ± 3.41	11.78 ± 3.09	0.02 *	0.39

SD: standard deviation; ROM: range of motion. * Significant differences between the conditions (*p* < 0.05).

## Data Availability

Not applicable.
